# The Food We Eat

**Published:** 1855-06

**Authors:** 


### THE FOOD WE EAT.

Men, women, children and cattle have been within a year past, and are now, starving for the want of necessary food, in a country too, capable of feeding abundantly a population fifty times greater than at present. It is stated that in Polk and Floyd counties, Georgia, there is such a scarcity of provisions, that many of the families are almost starving, and public meetings are called, to adopt measures of relief, in this year of 1855.

This state of things is attributed by some, to the want of rain or other unpropitiousness of season; by others to the high price of provisions, dependent on the European war. But reflection will perhaps convince all that the real reason is, too many people are trying to get along without work—to make money by speculation. Too many parents consider labor degrading, and consequently push their children into the professions, into salaried positions, when in reality they are far better adapted to the plow. Manual labor everywhere merits respect and honor. I have never pulled off my hat to man or woman born; but I do instinctively raise my hand to the brim, when I pass an iron foundry and see the men all blackly begrimmed, patiently working hour after hour in sweat and heat and dirt, and for a remuneration too, which barely covers the necessities of life for them and theirs; showing, however, they prefer thus to live and drag out an existence of toil, rather than by questionable practices, by gaming, by going in debt through false pretences, then failing full-handed, live the remainder of their days in idleness and ease. No man of reflection can help respecting such men, any more than he can help looking with contemptuousness on the well dressed loafer, or the aristocratic spendthrift who would not care to be seen talking to the toil-worn workman. And yet although we all know this, we turn right around and bring up our children to a mode of life which exempts them from manual labor. Thus it is, that people are starving in this most fruitful portion of the habitable globe. Not because of the potatoe rot, or from want of rain and fruitful seasons, but from the inconsistencies of parents in the middle and upper classes of society.

But the evil is upon us, and we want present relief; and to that end I propose offering some suggestions on the true economy of food. If we have not a good deal of it, and have but little money to purchase, the obvious plan should be to ascertain what kinds of food are most nourishing and cost least; and what are the most economical methods of preparation. For many of the facts I shall use, I am indebted to that useful and ably conducted weekly, the "American Agriculturist," published in New York, by Allen & Co., at two dollars a year; and also to the *Scientific American*, which needs no commendation—for it commends itself weekly to its tens of thousands of prepaying subscribers.

The most nutritious articles of food are meats and the cereals: that is, corn, wheat, oats, rye, &c. Vegetables are the least nutritious, and in cities especially, the most expensive; such as turnips, cabbages, parsnips and the like. Vegetables then being expensive and least nutritious, and as meats are not used by many from principle, and by a larger number from want of money, and moreover, as it ought not to be eaten but once in a day except by those who work hard, I will confine this article mainly to the consideration of the cereals and their preparation, of which the chief is

GOOD BREAD: AND HOW TO MAKE IT.

To make good wheaten bread, take a handful of hops, pour on three pints of water; boil, strain, and to the liquor add three large potatoes, after they have been washed, pared and grated, being stirred in while the liquor is boiling; then add one table-spoonful of salt, one tea-cupful of sugar or molasses, and thicken with a spoonful of flour; pour it out, and when cool enough, add yeast sufficient to rise it; when light, put it in a cool place, for use.

Next, pare and cut two quarts of potatoes, boil them in water enough to mix one gallon of sponge; when well boiled, wash and strain through a cullender, stir in flour while hot; when cool enough, stir in a tea-cupful of yeast—then set to rise; next morning make up your bread in the usual way; when light, mould it into loaves and let it stand until fit to be put into the oven. After it is baked, lay it on a wooden shelf to cool; when cooled, wrap it up in a cloth of some kind to keep it from drying and becoming hard.

### TO MAKE CORN BREAD.

Being a native of the land of "corn dodgers," I speak of what I have seen. My mother used to say that saleratus was poison—so it never appeared on our table in any form. The real, genuine, original "corn bread" is made as follows:—

Sift the Indian or corn meal, so as to be sure there are no bugs, crickets, spiders, sticks, nails, pebbles, &c. in it. By the way, our flour was sifted also. Stir as much water in this coarse sifted meal as will just make it stick together; take up a double handful, lay it over on one hand, flattening it a little with the other; lay it in the skillet, having been first greased a little. Usually, three of these handfuls fill the skillet, one "dodger" being laid beside the other; place the skillet on the hot coals and ashes, put on the lid, and cover it with the same, (from wood.) and when baked sufficiently, place them immediately on the table, in the shape of three *real* "pones" of bread; open one side-ways, put in a lump of fresh grass butter, and eat *ad libitum.* This is the way the negroes make it for themselves. The whites prefer to use milk instead of water, adding butter and eggs before cooking. Either way, the bread is delightful. But it cannot be made thus in the East, because our millers will persist in grinding it so fine, that it mixes into a paste, and in baking, becomes hard as a rock, unless it is puffed up and poisoned with saleratus, soda, cream of tartar, or some other physic.

### HOMINY

Is another cheap, nutritious and delightful article of food. Having been raised on "Hog & Hominy," I speak from the card. There is virtue in the original hominy mill; so I will begin at the beginning, and tell all about it. "Our John" was a manufacturer of hominy mills, and was great on wooden brooms, ("scrub brooms," as we called them;) and here, I

almost drop a literal tear to his memory, as he has just passed away, faithful in his old age to the last hour; for he was drowned in some unaccountable manner, while watering the plow horses. We never felt any hesitancy "a long time ago," in entrusting to him any amount of money; a more faithful, honest-hearted fellow never lived, and I trust he may look down and see by these lines, that he is not unhonored and forgotten.

But the hominy mill: A log was cut, four feet long and from twenty to thirty inches across. It was set on end, a fire kindled on the top, and kept burning until it was burned out in the shape of a bowl, large enough to hold a peck or more. This was called the "*hominy block;*" a few handfuls of corn, in the grain, were put in at a time, and with a wooden pestle, were pounded, until each grain was broken into four or five pieces. The chaff was blown off; some of it was placed in an earthen vessel, with just enough water to cover it, and let stand all night; early in the morning it was put on to boil, and kept boiling slowly, all day, stirring it occasionally, and adding water, just enough to keep it from burning. It may then be placed on the table, to be seasoned and eaten as each one may fancy. Some put salt on it; others butter, others molasses, others eat it with fresh milk. It is as good cold as warm. If any is left, it may be made into cake next morning, and warmed up with a little fat or butter. Nothing better, safer, cheaper and more nutritious, can be eaten, and it is wonderful, that in the great scarcity of provisions at this time, more of it is not used. It is as good with sugar or molasses, as in any other way, and children will revel in it thrice a day for weeks together; and sugar too is the only article of human consumption that is cheap at this time, as far as I remember; and more, it is very highly nutritious, as well as healthful. And as corn may be easily kept from one year's end to another, a poor man may snap his fingers triumphantly at beef steaks, 25 cents per pound, at two dollar potatoes, and thirteen dollar flour, by purchasing

| | |
|---|---|
| Eight bushels of shelled corn, say . . . | $8 00 |
| One barrel brown sugar, . . . . . | 11 00 |
| A hominy mill, . . . . . . | 7 00 |
| Total, | $26 00 |

Upon this outlay of twenty-six dollars, a good sized family, with a few other articles of trifling value, may subsist, may live, and will certainly fatten, for six months.

I here take occasion to say, that the *Scientific American*, of May 5th, 1855, page 268, describes a family hominy mill, to be obtained from the patentee, *B. Bridendolph*, *Clear Spring*, *Washington county*, *Maryland*.

This mill is so arranged, that it will grind corn and cob for cattle, or grind the corn grain into hominy; or finer, into meal. A hand power will make a bushel of hominy in an hour, while a horse power will grind from fifty to eighty bushels a day.

The two following tables, prepared by *Orange Judd*, the industrious editor of the *American Agriculturist*, are highly useful and instructive in their practical bearing on the economy of food

TABLE NO. ONE.

| 100 lbs. | Muscle forming elements. | Fat-forming elements. | Relative proportion of each. | Husky or woody-fiber. |
|---|---|---|---|---|
| Barley, . . . . . . . | 14 lbs. | 64 lbs. | 1 to 4½ | 15 lbs. |
| Beans, . . . . . . . . | 26 " | 42 " | 1 to 1¾ | 10 " |
| Beets, . . . . . . . | 2 " | 12 " | 1 to 6 | (?) |
| Buckwheat, . . . . . | 8 " | 54 " | 1 to 6¾ | 25 " |
| Carrots, . . . . . . | 1½ " | 10 " | 1 to 6⅔ | 3 " |
| Corn, . . . . . . . . | 12 " | 77 " | 1 to 6½ | 6 " |
| Oats, . . . . . . . | 17 " | 66 " | 1 to 4 | 20 " |
| Peas, . . . . . . . . | 24 " | 52 " | 1 to 2¼ | 8 " |
| Potatoes, . . . . . | 2 " | 19 " | 1 to 9½ | 4 " |
| Turnips, (field,) . . . | 1½ " | 9 " | 1 to 6 | 2 " |
| Do. Swedish, . . | 2¼ " | 12 " | 1 to 5½ | 2 " |
| Wheat flour, . . . . | 11 " | 79 " | 1 to 7 | |
| Wheat bran, . . . . . | 18 " | 6 " | 1 to ⅓ | 55 " |
| Cheese, (whole milk,) . | 28 " | 27 " | 1 to 1 | |
| Do., (skim-milk,) . | 45 " | 6 " | 1 to ⅛ | |

TABLE NO. TWO.

| | Cost. | | Muscle-producing elements. | Cost of muscle producing elements. |
|---|---|---|---|---|
| Barley, . . . . . . . | $1.50 | pr. bu. | 8.4 lbs. | 18c pr. lb. |
| Beans, . . . . . . . . | 2.50 | " | 16.6 " | 15 " |
| Corn, . . . . . . . | 1.10 | " | 6.7 " | 16⅔ " |
| Oats, . . . . . . . . | 68 | " | 5.2 " | 13 " |
| Peas, . . . . . . . | 2.00 | " | 14.3 " | 14 " |
| Potatoes, . . . . . . . | 1.50 | " | 1.6 " | 94 " |
| Turnips, . . . . . . | 50 | " | 1.2 " | 41 " |
| Flour, (fine,) . . . . . | 12.00 | per bbl. | 22.0 " | 54 " |
| Flour, (unbolted,) . . | 11.00 | " | 24.8 " | 44 " |

These tables will richly pay their patient and mature study; for their practical use will save many a dollar to the industrious poor. The estimates will be better understood by remembering that the *muscle-forming elements* means *strength*—the strength of labor—or you might better term it "labor power." For example, eighteen cents worth of barley gives one pound of *labor power.* A bushel of barley gives over eight pounds of *labor power.* We may make a comparison in parallel lines, to render it clear and memorable. At the prices above named,

| | | | | |
|---|---|---|---|---|
| One pound of labor-power from | potatoes, | costs | 94 | cents!! |
| " " " " " " | fine flour, | " | 54 | " |
| " " " " " " | unbolted flour, | " | 44 | " |
| " " " " " " | turnips, | " | 41 | " |
| " " " " " " | barley, | " | 18 | " |
| " " " " " " | corn, | " | 17 | " |
| " " " " " " | beans, | " | 15 | " |
| " " " " " " | peas, | " | 14 | " |
| " " " " " " | oats, | " | 13 | " |

After all, "Sawny" is not only a money-making machine, but a philosopher; he revels in oat meal porridge on principle—on the solid principle of its helping him to do the most labor at the smallest possible money cost. But Jonathan won't eat oats, and it isn't worth while to talk about it. So we fall back on beans in general; but being a Kentuckian in particular, I'll stick to the corn—that is, in theory. As to actual practice, I must say I do not particularly like corn bread. Our mother being an Englishman, used to say it tasted to her like sawdust; so we imbibed her prejudices, which remain to this day. But I have no doubt corn bread is good, judging from its universal appearance on western tables.

Beans then, as an article of food, are six times cheaper, at two dollars and a half a bushel, than potatoes at a dollar and a half a bushel; that is, give six times more substantial nutriment.

CABBAGE, not from the tailor's shop, but the product of mother earth, would, says the "*Country Gentleman,*" soon become the leading article of human food, if cooked thus:—Boil it by itself, in pure water, till cooked perfectly soft, then serve up, adding butter and salt to your liking, without vinegar or pepper." One pound of cabbage seed will yield 24,000 plants, and about eight thousand plants are required to an acre; with such productiveness, I accord with the New York Tribune, when it declares in an able article on "starvation prices," that it is the duty and interest of every man who owns a piece of land, wherever he can sow a bushel of seed, not to allow the spring to slip by without doing it.

### THE USE OF FRUITS.

While on the all important subject of eating, I may as well make a few suggestions as to their use, although this article is already long.

Some people have a perfect *phobia* of fruits—especially in summer time, when most abundant, most perfect and in their season. As there is no help for the ratiocinative capabilities of such folk, we will pass them by, and address our remarks to people of plain common sense; that happy class who have no kinks on either side of the skull.

Fruits and berries of every description, if properly used, are the great preventives of all summer diseases, of fevers, fluxes, head aches, side aches, neuralgias, blue devils, dumps didoes and desperations.

How?

Because their natural tendency is to prevent constipation, and by keeping the bowels soluble, that is, daily acting, they give an outlet to all febrile and bilious "humors," thus keeping the system cool, and carrying from it all its excess of blood. Our perversity takes everything in its season but fruits. Even a pig is tabooed in summer; but fruits we muss up, and distort with sugar, and molasses and spices, to be consumed in winter time, when we don't want any cooling off. But that is always the way with people of uncommon sense; so we folks who are fortunately lower down in the scale of practical life, may luxuriate in the greater abundance. I may be told here that General Taylor was killed by a dish of fruit, and so he was; and that multitudes of children in cities are destroyed by eating "*such trash*," as it is called, and so they are—*not;* for only rich people can afford to buy fruit at any season of the year, in large cities; and in the summer time they take their children out of town.

It was not the fruit that killed the honest-hearted old soldier; but it was the ice and cream he took with it, while the system was exhausted with heat and fatigue, consequent on the ceremonies attendant on laying the corner stone of the Washington monument, on the fourth of July. This might not have been sufficient, had he not within a short time after, while these articles were still but half digested, eaten a very hearty dinner, contrary to the express remonstrances of a friendly physician who was present.

Fruits and berries are healthy every day of the year, whether a man is sick or well; actual observation has established the fact, that fruit is medicinal even in diarrhœa, inasmuch as it has a curative effect, when properly used. It is a first truth in allopathic medicine, that in almost every disease, the bowels must be kept free; and that is the natural tendency of fruits and berries of every description. I know from actual observation, that there is not a more healthy class of people in the world, than the negroes who work in the cotton fields and sugar plantations of the south; to look at them working in the hot sun of 112° Fahrenheit, and breathing the clouds of dust which in a dry time arise from the use of the hoe, one would think that they would actually melt; but they neither melt nor die, but will work all day and go home at night, sing songs and "*dance juber*," by the hour—in which I have joined, and therefore am a competent witness; for in younger days, I delighted "immensely" to peer about and look—how look, reader? There are many ways of looking now a days: I did not look under or over or around things, but straight at them, and that is precisely the reason I know so much according to the unanimous opinion of *me ipsum*.

Well, what has a cotton plantation, which John Mitchell wanted so badly and didn't get—what has a cotton plantation and its "*hands*" to do with the healthfulness of fruit, the very thing they never see? That is true, but it is necessary to eke out copy, lest I should tell you so much important truth you cannot remember half of it. But let us go back and "*make the connection*," a thing which railroad companies and hungry hotel keepers do not always do, *on purpose*. I was saying, that in the hottest fields of the south, and under the hardest labor, the laborers thrive and shine—yes, literally shine, as any well-fed negro will do; well, these "hands" have two actions of the bowels daily, that is, I have questioned them on the subject, and they told me so. It is fair, then, to infer that a free state of the bowels in summer time is an attendant of sound robust health; all know that fruits have that tendency, and consequently they must be healthy. The banana of Cuba is the meat and bread, the all and all of the slave population; they can live wholly on that alone, as I have seen them do, for weeks together, and the banana is nearer in its nature to fruit than any thing else we know.

Now, reader, if I have not convinced you of the value of fruit in summer, just let it alone, and send your share to Forty-two Irving Place, New York, and I will receive it with many thanks, and cure up your throat and knock the consumption out of you for —— a consideration, that is, beside the fruit present. One poor fellow, two or three summers ago, kept me supplied with fruit all the season, more than I wanted, so I sent it around to friends: yet I didn't cure him, he died; but he didn't follow the directions, and of course I was not to blame; among the chief of these was, uniformly, *pay as you go*, but he forgot that, and perhaps that was the reason I did not cure him. But to come at once to the conclusion of the whole matter, it only remains to tell

### HOW TO USE FRUITS

In the summer time, so as to derive from them all those nutritious, delightful and health-giving influences, which a kind Providence intended doubtless should follow their employment. Fruits and berries should be ripe, fresh, perfect—should be eaten, the earlier in the day the better, not later certainly than three o'clock in the afternoon—should be eaten alone, unless with loaf sugar, not within two hours of eating any thing else, and drinking nothing within half an hour of so eating them.

The reason for these restrictions I cannot here add, after such a long article; but for the present, the reader must search for himself; in the mean while, let him use fruits and berries as directed, and he may do it without restriction as to quantity, and will find them to be among the most delicious, as well as the most healthful and invigorating aliments in all nature.

